# Semen IgM, IgG1, and IgG3 Differentially Associate With Pro-Inflammatory Cytokines in HIV-Infected Men

**DOI:** 10.3389/fimmu.2018.03141

**Published:** 2019-01-23

**Authors:** Thevani Pillay, Parveen Sobia, Abraham Jacobus Olivier, Kapil Narain, Lenine J. P. Liebenberg, Sinaye Ngcapu, Mesuli Mhlongo, Jo-Ann S. Passmore, Cheryl Baxter, Derseree Archary

**Affiliations:** ^1^Centre for the AIDS Programme of Research In South Africa (CAPRISA), University of KwaZulu-Natal, Durban, South Africa; ^2^Institute of Infectious Diseases and Molecular Medicine (IDM), University of Cape Town, Cape Town, South Africa; ^3^Department of Medical Microbiology, University of Kwazulu-Natal, Durban, South Africa; ^4^National Health Laboratory Service (NHLS), Cape Town, South Africa; ^5^Division of Medical Virology, Department of Pathology, University of Cape Town, Cape Town, South Africa

**Keywords:** semen, HIV, HIV-specific antibodies, immunoglobulins, cytokines, genital inflammation

## Abstract

Genital inflammation significantly increases the risk for HIV infection. The seminal environment is enriched in pro-inflammatory cytokines and chemokines. Here, we investigated the interplay between semen cytokines and humoral immunity to understand whether the characteristics of semen antibodies are associated with genital inflammation. In 36 HIV-infected and 40 HIV-uninfected mens' semen, HIV-specific antibodies (gp120, gp41, p66, and p24), immunoglobulin (Ig) subclasses, isotypes and cytokines, using multiplex assays, were measured. Semen IgG1, IgG3, and IgM were significantly higher in HIV-infected compared to HIV-uninfected men (*p* < 0.05). In HIV-uninfected men, pro-inflammatory cytokines IL-6, IL-8, and MCP-1 significantly correlated with IgG1 and total IgG (IgG1+IgG2+IgG3+IgG4) (both *r*≥0.55; *p*≤0.001). Total IgG in HIV-infected men correlated to HIV-specific antibodies in the semen irrespective of antiretroviral (ARV) use. In HIV-infected, ARV-treated men, p66 and gp41-specific antibodies were inversely correlated with IL-6 and MIP-1α (both *r*≥−0.65, *p*≤0.03). In HIV-infected, ARV-naïve men, p24 and gp120-specific antibodies correlated significantly with pro-inflammatory TNF-α (*r*≥0.44, *p*≤0.03), while p24 antibodies correlated significantly with chemokine MIP-1β (*r* = 0.45; *p* = 0.02). Local cytokines/chemokines were associated with the mucosal-specific Ig subclasses which likely effect specific antibody functions. Together, these data inform on mucosal-specific immunity that may be elicited in the male genital tract (MGT) in future vaccines and/or combination HIV prevention strategies.

## Introduction

Globally, HIV transmission through sexual intercourse remains the predominant cause of new infections. Several factors influence the risk of HIV acquisition between sexual partners such as the viral loads of transmitting partners (1), other sexually transmitted infections (STIs) (2, 3), the use of pre-exposure prophylaxis (PrEP), partner use of antiretroviral (ARV) treatment in serodiscordant couples (4, 5), the number of sexual partners, male circumcision (6–8) and anal intercourse (9). Genital inflammation has come under the spotlight as a factor that significantly increases HIV-acquisition risk.

Genital inflammation, defined as a specific profile of inflammatory cytokines (10–12), increased T-cell activation and HIV target cell recruitment (13, 14), has been identified as a significant risk factor for HIV acquisition in women (10, 15, 16). Whether the definition of genital tract inflammation is gender biased, remains less well-described. Olivier et al. (17), Linge et al. (18) and Politch et al. (19) reported consistently higher concentrations of inflammatory cytokines in the semen than the blood, irrespective of HIV-infection status (20), reflecting a persistent and primed state of immune activation conducive to HIV infection. Studies confirmed that the cytokine and chemokine seminal plasma milieu supports active viral replication through ongoing activation of target CD4 T cells *in situ* (13, 14, 20). Higher levels of inflammatory cytokines were associated with increased HIV shedding in the genital tract, increasing the risk of transmission to sexual partners (21). In HIV-infected men on ARV treatment, systemic viral suppression has not always reflected full viral suppression in the seminal compartment, possibly due to semen virus being refractory to the effects of ARVs (22–24). HIV acquisition can indeed still occur in healthy individuals on PrEP, despite good adherence and high blood levels of active drug (25, 26), highlighting the complexity of HIV acquisition secondary to local mucosal-specific virus traits.

The profiles of local cytokines can also directly affect the immunoglobulin (Ig) types and subclasses, and therefore functions of the humoral response (27), likely even at the mucosae. During acute HIV infection, IgM is the first isotype to respond. Studies have demonstrated that IgM directly dampened inflammation through reducing T-cell activation/proliferation and reduced production of certain pro-inflammatory cytokines such as TNF-α, IFN-γ, and IL-17. IgM demonstrated direct interaction with co-receptors CCR5 and CXCR4, thereby preventing chemokine or HIV-1 virion binding (28–30). Passively transferred recombinant monoclonal IgM completely protected non-human primates after mucosal SHIV challenge suggesting that the pentameric IgM through cross-linking is highly efficient at virus capture (31). Previous studies have shown that HIV-specific antibodies present in semen, tend to be IgG rather than IgA dominant (32, 33). At the IgG subclass level, IgG1 and IgG3 subclasses demonstrated higher affinity for the neonatal Fc receptor (FcRn) than IgG2 and IgG4 (34). IgG3 and IgG1 are particularly efficient in driving direct neutralization (35, 36), have higher affinity for FcγRIIIa on natural killer (NK) cells to exert cytotoxic and phagocytic functions (37–40). Env V1V2-specific IgG3 in particular, shown to drive ADCC, correlated with lower HIV infection risk in the RV144 vaccine trial compared to the VAX003 trial (38, 41). Furthermore, IgG subclass profiles and HIV-specific antibody classes and titres significantly and directly correlated between the blood and semen (42, 43). In semen, whether the genital inflammation affects the various antibody classes, specificities and titres, remains less well-defined.

We investigated whether male genital tract (MGT) inflammation influenced the antibody subclasses and HIV-specific antibody titres in seminal fluid from HIV-uninfected and HIV-infected men who were either ARV-treatment experienced or were ARV-treatment naïve. We hypothesized that the characteristics of the humoral immune responses would be significantly influenced by the local cytokine milieu in the semen of HIV-infected and uninfected men.

## Materials and Methods

### Study Participants and Sample Collection

Semen and blood samples were donated by 36 HIV-infected and 40 HIV-uninfected men, enrolled at the Empilisweni Clinic in Athlone, Cape Town, South Africa (21). All men voluntarily donating semen gave written informed consent, and the Research Ethics Committee of the University of Cape Town approved all aspects of the study. Ejaculates were collected following voluntary self-masturbation and processed according to the method described by Oliver et al. (17). Seminal plasma was stored at −80°C. CD4 T cell counts in peripheral blood were performed using Flow CARE PLG kits (Beckman Coulter, Inc., Brea, CA), according to the manufacturer's protocol.

### Plasma and Semen HIV Viral Load Quantification

NucliSENS EasyQ HIV-1 (version 2.0, BioMérieux SA, Lyon, France) was used to quantify plasma and semen HIV-1 RNA concentrations (copies/mL) with a lower limit of detection (LLD) of < 70 copies of HIV-1 RNA/mL and a linear range of detection up to 10 × 10^6^ copies of HIV-1 RNA/mL.

### HIV-1-Specific Binding Antibody Multiplex Assay (BAMA)

Antibodies directed against four HIV proteins were measured using a customized HIV-1 binding antibody multiplex assay (BAMA), as previously described (44–47). The antigen panel included the following HIV proteins: gp120 (Jena Bioscience, Germany), gp41 recombinant HIV-1 MN (ImmunoDX, USA), p66 HIV-1 R (Protein Sciences Corporation, USA), and p24 HIV-1/Clade B/C (Immune Technology, USA). Antibody titres were reported as a mean fluorescent intensity (MFI). As negative controls, beads with no antigen and normal human serum were utilized, which were subtracted from MFIs for that of the HIV-positive semen samples. All assays were conducted using good clinical laboratory practice principles, in which positive controls were tracked using Levy-Jennings charts as described previously (45, 47). Criteria for considering an antibody-antigen interaction positivity in this assay were determined using a panel of seminal plasma samples from 40 HIV-uninfected individuals (MFI ± 3 standard deviations) and a first-level cut-off of at least 100 MFI thereafter determined a positive result that was HIV-antigen specific.

### Total, Isotype and HIV-Specific Immunoglobulin Quantification

To normalize for the inter-subject variation in seminal plasma recovery, IgG1, IgG2, IgG3, IgG4 (total IgG), IgM, and IgA were quantified using a 6-plex antibody isotyping kit (Bio-Rad Laboratories, Inc; Hercules, Ca, USA), according to the manufacturer's instructions. MFIs were determined using a Bio-Plex 200 suspension array system. Seminal plasmas were diluted at 1:20 in sterile phosphate buffered saline (PBS) to ensure that MFIs for both the isotype and HIV-specific antibody multiplex assays were detected in the linear range of the standard curve.

Total IgG is calculated by the sum of IgG1, IgG2, IgG3, and IgG4 (ng/ml). HIV-specific IgG activity in seminal fluid was calculated as a ratio of each of each HIV-specific IgG from the BAMA assay (Log_10_ MFI^*^dilution factor), divided by total IgG (ng/ml) and adjusted for the dilution factor [Log_10_ (MFI/ng/ml^−1^)], this value reflects specific activity. A second level cut-off for specific activity, based on 40 HIV-uninfected men recruited for this study were applied to determine a positive response in the seminal fluid. Values falling below a detectable specific activity cut-off were included in the analysis and assigned a value of the average specific activity cut-offs for each HIV-specific IgG in the 40 HIV-uninfected men.

### Cytokine Measurements in Semen

The concentrations of 20 cytokines were measured in semen and blood of HIV-infected and HIV-uninfected men using high-sensitivity human and human cytokine Milliplex MAP kits (Millipore Corporation, St. Charles, MO), as previously described (21). These included interleukin (IL) −1β, IL-2, IL-6, IL-7, IL-12p70, granulocyte macrophage colony-stimulating factor (GM-CSF), interferon-gamma (IFN-γ), and tumor necrosis factor-alpha (TNF-α), with the kit sensitivity ranging from 0.05 pg/mL to 0.46 pg/mL for each cytokine. The following analytes were measured with the human cytokine kit: IL-1α, IL-8, IL-10, IL-12p40, IL-15, Eotaxin (CCL11), Fractalkine (CX3CL1), granulocyte colony-stimulating factor (G-CSF), monocyte chemotactic protein (MCP-1; CCL2), macrophage inflammatory protein (MIP)-1α (CCL3), MIP-1β (CCL4), and regulated upon activation normal T cell expressed and secreted (RANTES; CCL5), the kit sensitivity ranged from 0.2 to 10.5 pg/mL for each cytokine. Semen samples were thawed and filtered by centrifugation using 0.2 μM cellulose acetate filters (Sigma), prior to cytokine/chemokine measurements. Data were collected using a Bio-Plex Suspension Array Reader (Bio-Rad Laboratories Inc., Hercules, CA) and Bio-Plex manager software (version 4). Cytokine concentrations below the lower limits of detection were reported as the midpoint between the lowest concentration and zero for each cytokine measured, as previously described (10, 21, 48).

### Statistical Analyses

All graphs were drawn using GraphPad Prism 7. One-way ANOVA analyses were performed to compare the titres of the Ig isotypes and subclasses of antibodies and the HIV-specific antibody activities between the HIV-infected ARV treated, HIV-infected ARV naïve and HIV-uninfected men. β-estimates were reported as 1 pg/ml increase in cytokines and 1 ng/ ml increase of Ig. Spearman Rho test was used for correlations between HIV-specific antibodies and total Ig. HIV-specific activities and IgG values were log transformed to ensure normality and a *p* < 0.05 was considered significant. Variance inflation factor (VIF) analysis, was performed before an initial step-wise linear regression, to control for multi-collinearity between independent variables. In HIV-uninfected men, 10 of the 20 cytokines qualified for a VIF threshold of 5, while in HIV-infected men, 13 of the 20 cytokines measured, qualified for the same. The resulting model was further adjusted with Cook's distance to identify the most influential observations. Statistical analyses were performed using R (statistical programming language).

## Results

### Baseline Demographic Characteristics

Semen obtained from 36 HIV-infected and 40 HIV-uninfected black heterosexual South African men, as previously described by Olivier et al. (21), were used to assess associations between the inflammatory cytokine milieu in semen during HIV infection and local mucosal antibody titer or specificity. Of the 36 HIV-infected men, 11 (30.6%) were receiving ARV (HIV^+^ARV^+^) at the time of study. Twenty-five HIV -infected men (69.4%) were ARV naïve (HIV^+^ARV^−^), the majority having detectable HIV in their semen, with a median semen viral load of 2,150 RNA copies/mL (range [LDL-135,000] RNA copies/mL) (Table 1). The majority of HIV^+^ARV^+^ men had a semen viral load below the limit of detection level (*n* = 9), with only two men with viral loads of 414 and 60,202 RNA copies/mL. Twenty-one of the 40 (52%) HIV-uninfected men reported having an HIV-infected female partner (discordant negative) while 14 (35%) had HIV-uninfected female partners (concordant negative).

**Table 1:** Cohort characteristics of the HIV-infected and HIV-uninfected men.

**Characteristic**	**HIV^**−**^**	**HIV^**+**^ARV^**−**^**	**HIV^**+**^ARV^**+**^**
***N***	40	25	11
**Age, years (median[Range])**	44 [23–58]	40 [30–54]	44 [39–47]
**PARTNER STATUS (n/N):**			
Concordant negative	14/40	–	–
Concordant positive	–	13/25	5/11
Discordant negative	21/40	–	–
Discordant positive	–	10/25	6/11
Partner status unknown	5/40	2/25	–
CD4 count, cells/mm3 (median[Range])	–	404 [137–737]	264 [179–748]
Plasma viral load, RNA copies/mL (median [Range])	–	11000 [LDL-300,000]	LDL[LDL-880]
Semen viral load, RNA copies/mL (median [Range])	–	2150 [LDL-135,000]	LDL[LDL-60200]
Number of men with detectable HIV RNA in semen n/N	–	19/25	2/11

*LDL, lower than detection level <70 HIV-1 RNA copies/mL*.

### Total Semen IgG1, IgG3 and IgM Were Higher in HIV^+^ Than HIV^−^ Men

**Figure 1:**
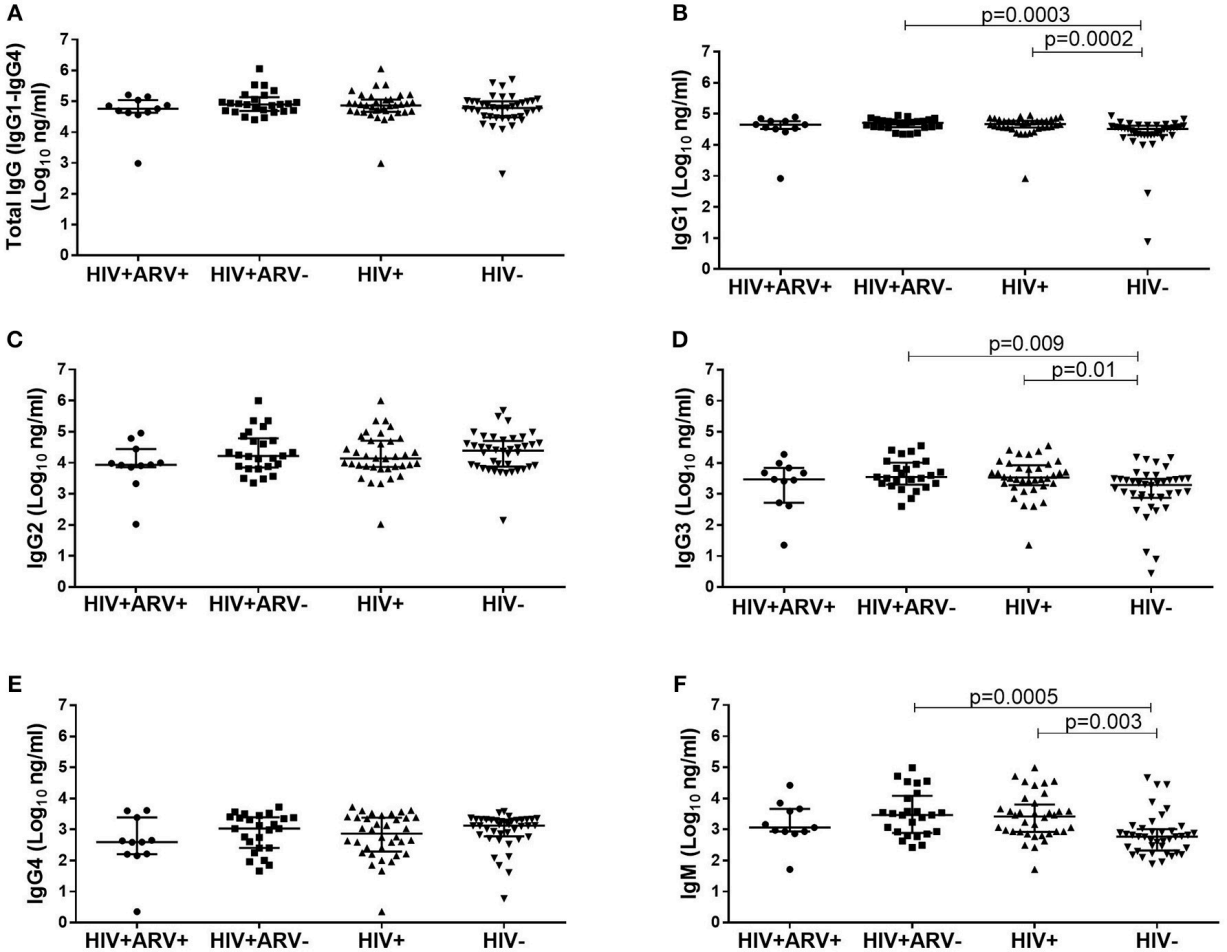
Comparison of the total IgG (IgG1–IgG4) in semen from **(A)** HIV^+^ARV^−^ (*n* = 25), HIV^+^ARV^+^ (*n* = 11), HIV^+^ (*n* = 36), and HIV^−^ men (*n* = 40), including IgG antibody subclasses; **(B)** IgG1, **(C)** IgG2, **(D)** IgG3,**(E)** IgG4, and isotype; **(F)** IgM. Each data point represents an individual sample, with medians and interquartile ranges. ANOVA analysis was used to compare multiple groups and *p* < 0.05 were considered statistically significant.

To evaluate the impact of HIV infection and ARVs on total IgG profiles in semen, quantifications of total IgG (IgG1, IgG2, IgG3, and IgG4) were performed (Figure 1). Total IgG titres were similar between the groups, although individual subclass differences were evident (Figure 1A). HIV^+^ men had significantly higher median titres of IgG1 [median 4.67 log_10_ ng/ml (IQR 4.56–4.78); *p* = 0.0002], IgG3 [median 3.52 log_10_ ng/ml (IQR 3.28–3.92); *p* = 0.01] and IgM [median 3.42 log_10_ ng/ml (IQR 2.92–3.80); *p* = 0.003] compared to HIV^−^ men [IgG1 median 4.51 log_10_ ng/ml (IQR 4.32–4.62), IgG3 median 3.29 (IQR 2.87–3.49), IgM median 2.77 (IQR 2.32–3.00)] (Figures 1B,D,F) IgG2 and IgG4 were similar between the groups (Figures 1C,E). Additionally, these differences remained significant after the removal of the data for the outliers.

### HIV-Specific Activity Is Similar in HIV^+^ Men Irrespective of ARV Usage

HIV-specific activities for all four antibody-specificities were unsurprisingly higher in HIV^+^ compared to HIV^−^ men (*p*≤0.0002) (Supplementary Figure 1). The HIV-specific activities were similar in HIV^+^ men using ARVs to those naïve to ARV treatment. While the majority of the HIV^+^ARV^−^ men showed detectable HIV-specific activities, seven of the 25 men had undetectable specific activities to p24, p66, gp41, and gp120 (Supplementary Figures 1A–D). In the HIV^+^ARV^+^ men, only one individual had undetectable specific activities to p24, p66, gp41, and gp120. One of the two HIV^−^ men had detectable p24 and p66-antibody specific activities (Supplementary Figures 1A,B).

### Total IgG Concentrations Correlated With HIV-Specific Antibodies in Semen During HIV Infection

**Figure 2:**
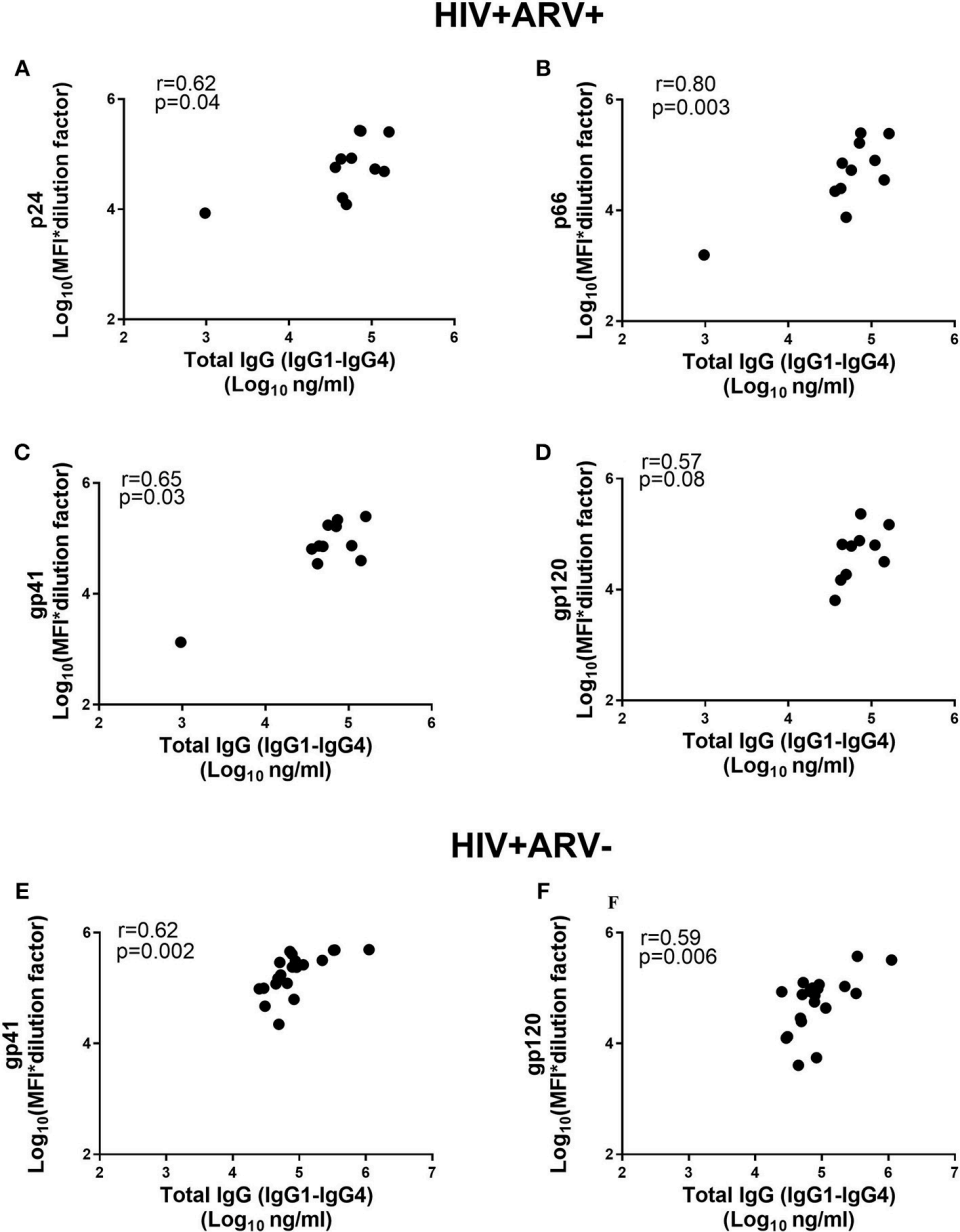
Comparison of total IgG (IgG1–IgG4) antibodies (Log_10_ ng ml^−1^) and MFIs for the HIV-specific IgGs [Log_10_ (MFI^*^dilution factor)] in HIV^+^ARV^+^ (*n* = 11) men for **(A)** p24, **(B)** p66, **(C)** gp41 **(D)** gp120, and HIV^+^ARV^−^ (*n* = 25) for **(E)** gp41 and **(F)** gp120.

Despite the similarities of the HIV-specific antibody detectability and specific activities in the ARV-experienced and ARV-naïve men, we investigated the relationship between the HIV-specific antibodies and total IgG (IgG1+IgG2+IgG3+IgG4) in both groups. There were significant and positive correlations observed for p66 (*r* = 0.80; *p* = 0.003, Figure 2B) and gp41 (*r* = 0.65; *p* = 0.03, Figure 2C) and more moderate associations for p24 (*r* = 0.62; *p* = 0.04) and gp120 (*r* = 0.57; *p* = 0.08) (Figures 2A,D). In the HIV^+^ARV^−^ group, significant associations were found for gp41 (*r* = 0.62; *p* = 0.002, Figure 2E) and gp120 (*r* = 0.59; *p* = 0.006, Figure 2F), whereas no significant correlations for p24 and p66 were observed in relation to total IgG.

### Semen Cytokines Concentrations Were Increased in HIV-Infected Men

The differences in soluble cytokine profiles in semen from HIV^+^ (*n* = 36) and HIV^−^ (*n* = 40) men was compared to determine if the differences were still present despite the smaller sample size from the original study (21). As previously reported, the majority of cytokines remained similar between the two groups. HIV^+^ men showed significantly higher MIP-1α [median 0.95 log_10_ pg/ml (IQR 0.47–1.60)] and fractalkine [median 2.85 log_10_ pg/ml (IQR 2.43–3.12)] compared to HIV^−^ men (*p* = 0.03 and *p* < 0.0001) respectively. Whereas, IL-10 [median 0.87 log_10_ pg/ml (IQR 0.18–1.32)] and eotaxin [median 1.34 log_10_pg/ml (IQR 0.9–1.58)] remained significantly higher in HIV^−^ compared to HIV^+^ men (*p* = 0.009 and *p* < 0.0001), respectively. No differences were observed between the HIV^+^ARV^+^ vs. the HIV^+^ARV^−^ men (data not shown).

### Associations Between Cytokines and HIV-Antibody Specific Activities in Semen

**Figure 3:**
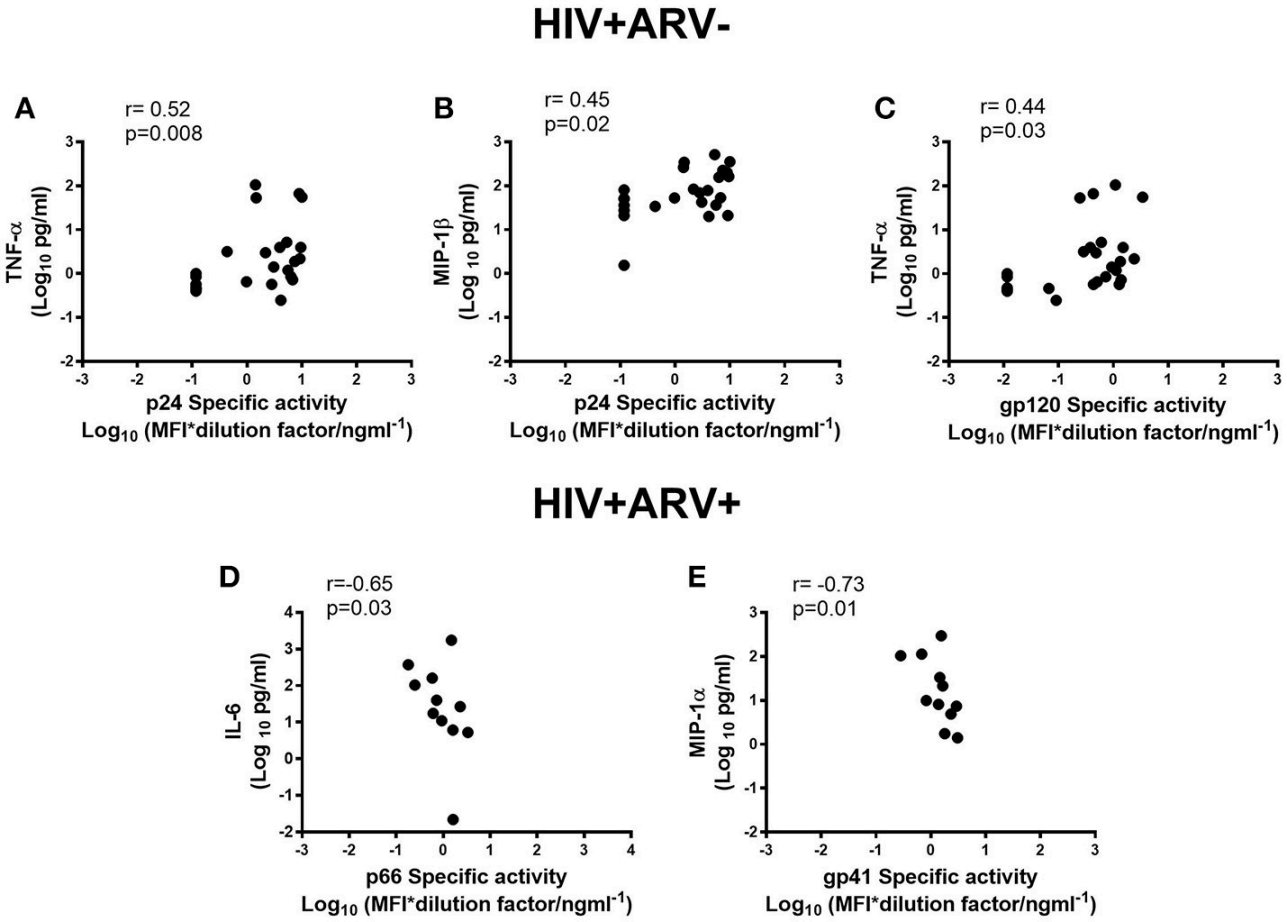
Associations of semen cytokines-TNF-α and MIP1-β with p24 specific activities **(A,B)**, and TNF-α with gp120 specific activity (Log_10_ MFI^*^dilution ng ml^−1^) **(C)** in HIV^+^ARV^−^men. Association of IL-6 with p66 specific activity **(D)** and MIP-1α with gp41 specific activity **(E)** in HIV^+^ARV^+^ men.

The levels of HIV-specific antibodies were also investigated to determine whether there was an association with inflammatory cytokine responses locally in semen, as previous studies have suggested that local inflammation influences viral loads in semen (21). The concentrations of TNFα in semen correlated with p24- (*r* = 0.52; *p* = 0.008) and gp120-specific activities (*r* = 0.44; *p* = 0.03) (Figures 3A,C) in HIV^+^ARV^−^ men. Similarly, MIP-1β concentrations in semen correlated with p24-specific activity (*r* = 0.45; *p* = 0.02) (Figures 3B; Supplementary Table 1). In contrast, in HIV^+^ARV^+^ men, there were significantly strong but negative correlations between p66-specific activity and IL-6 (*r* = −0.65; *p* = 0.03) and, gp41 and MIP-1α (*r* = −0.73; *p* = 0.01) (Figures 3D,E; Supplementary Table 2).

### Relationship Between Ig Subclass, Isotypes, and Cytokines in Semen

**Figure 4:**
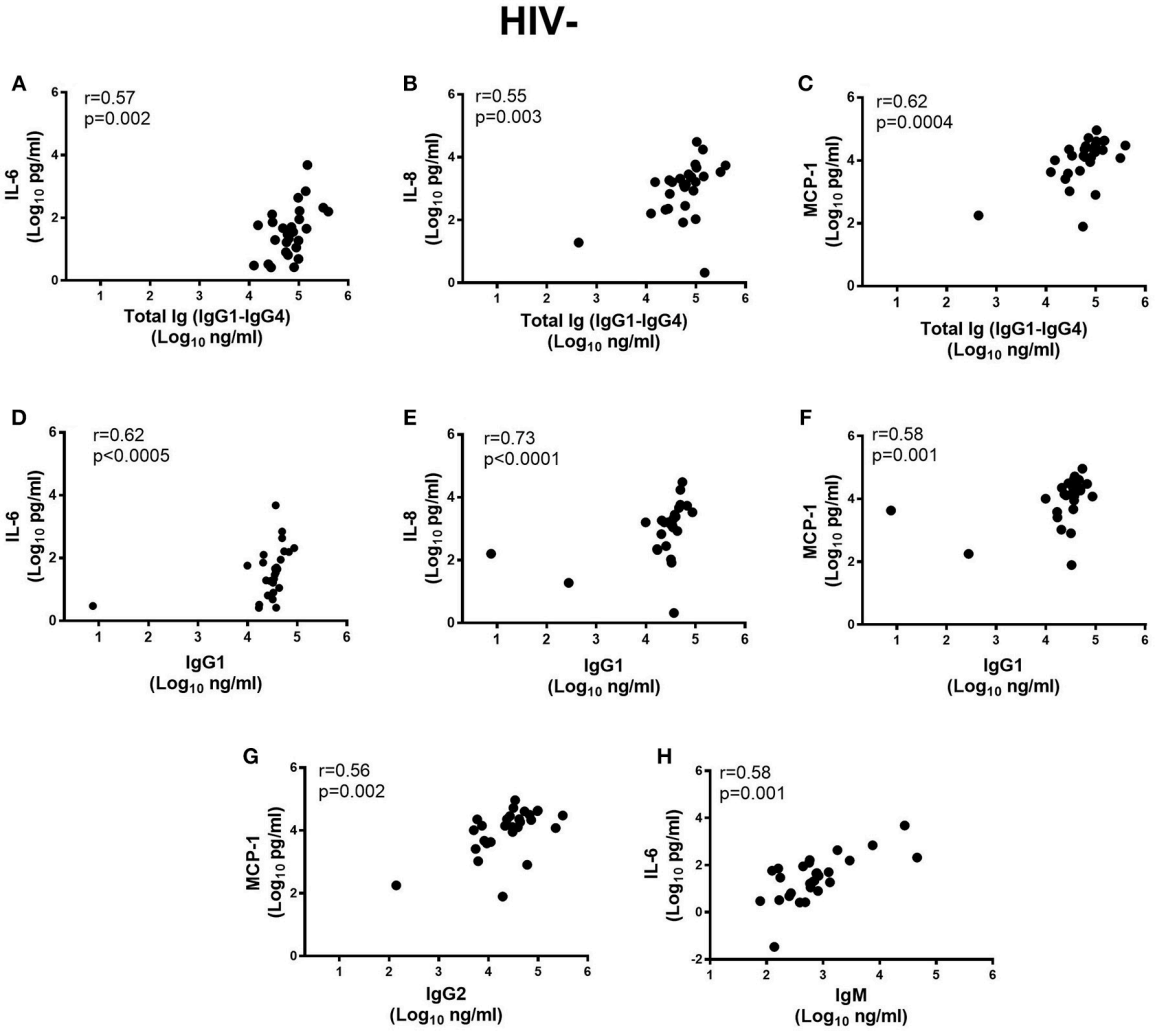
Correlations of semen cytokines IL-6, IL-8, and MCP-1 with **(A–C)** total Ig (IgG1–IgG4), Ig subtypes **(D–F)** IgG1, **(G)** MCP-1 with IgG2 and **(H)** IL-6 with IgM in HIV uninfected men (*n* = 28). To remain significant after Bonferroni multiple comparison adjustment, a *p* ≤ 0.0025 was required.

**Figure 5:**
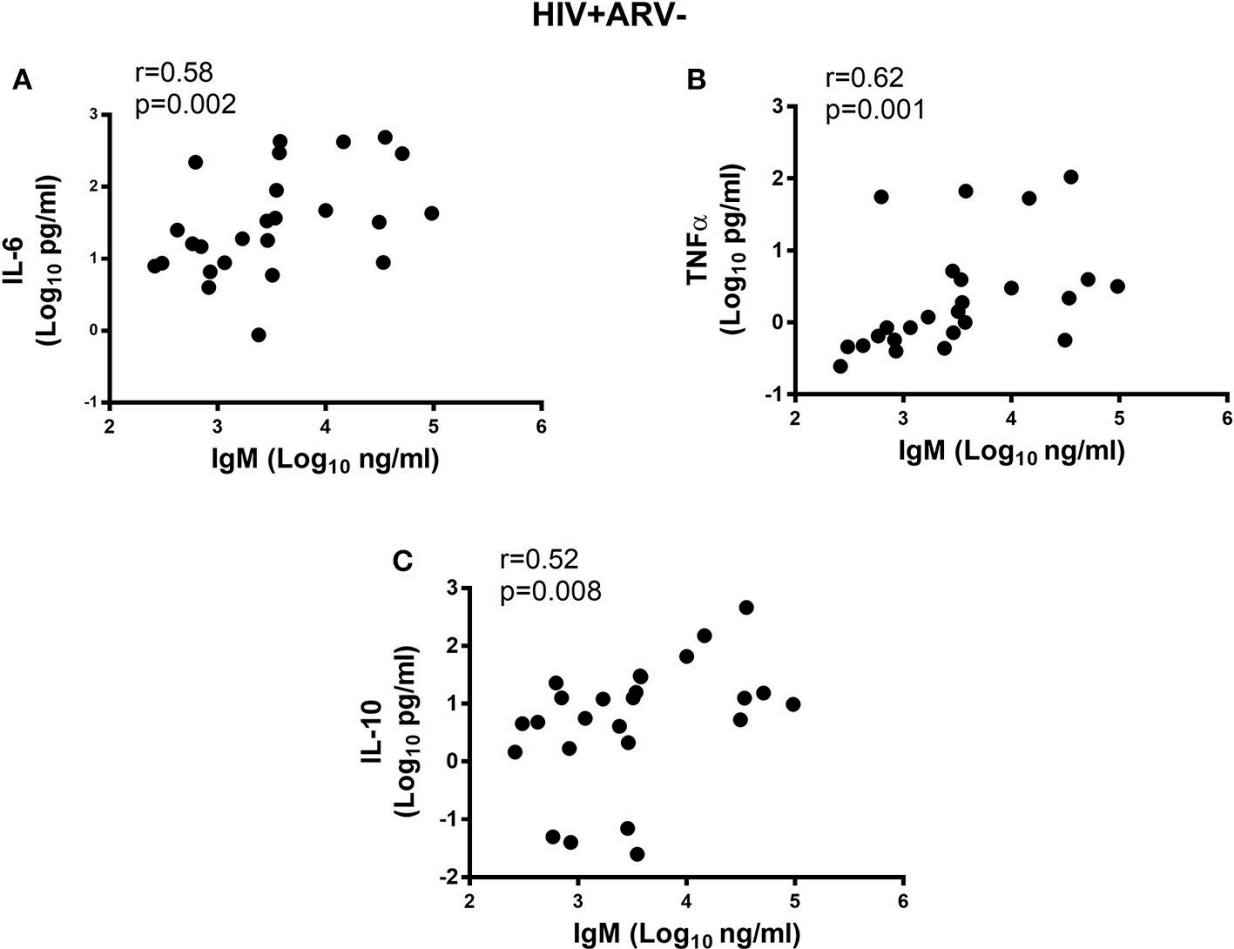
Correlations of semen cytokines with the IgM (Log_10_ ng ml^−1^) and **(A)** IL-6, **(B)** TNFa and **(C)** IL-10 in HIV^+^ARV^−^ men. All significant associations withstood Bonferroni multiple comparison adjustment.

Next, we determined if inflammatory cytokine milieu in the semen was associated with altered Ig subclass profiles. In HIV^−^ men, pro-inflammatory IL-6 (*r* = 0.57; *p* = 0.002) and IL-8 (*r* = 0.55; *p* = 0.003) and chemotactic MCP-1 (*r* = 0.62; *p* = 0.0004) correlated positively with total IgG titers in semen (Figures 4A–C) and IgG1 (*r* = 0.62; *p* < 0.0005, *r* = 0.73; *p* < 0.0001, *r* = 0.58; *p* = 0.001, respectively) (Figures 4D–F). Furthermore, IgG2 correlated positively with MCP-1 concentrations in semen (*r* = 0.56; *p* = 0.002) and IgM with IL-6 concentrations (*r* = 0.58; *p* = 0.001) (Figures 4G,H), after adjusting for multiple comparisons. For the HIV^+^ARV^−^ men, semen concentrations of IL-6 (*r* = 0.58; *p* = 0.002), TNF-α (*r* = 0.62; *p* = 0.001), and IL-10 (*r* = 0.52; *p* = 0.008) correlated significantly with IgM titres (Figures 5A–C).

Regression analyses were conducted to determine the impact of MGT inflammation on antibody isotypes. Using this regression model, IL-15 levels were significantly associated with IgG1 and IgG2 concentrations in HIV^−^ men; for every 1 pg/ml increase in IL-15 in the semen, IgG1 and IgG2 increased by 270.5 ng/ml (*p* = 0.007) and 1126.3 ng/ml, respectively (*p* = 0.004) (Table 2). Similarly, eotaxin showed significant, positive associations with IgG3, IgM and IgA (*p* = 0.001, *p* = 0.01, and *p* = 0.004, respectively) (Table 2). In contrast IL-1α, exhibited a negative association with IgM (*p* = 0.04) (Table 2).

**Table 2:** Beta estimates reflecting the association between semen cytokines and Ig subclass and isotype in HIV-uninfected men.

**Cytokine**	**Beta Estimate ng/ml**	**Confidence Interval**	***P*-value**
		**IgG1**	
IL-15	270.5	79.6 to 461.4	0.007
		**IgG2**	
IL-15	1126.3	404.9 to 1847.8	0.004
		**IgG3**	
IL-8	0.5	0.3 to 0.6	<0.001
Eotaxin	1.1	0.5 to 1.6	0.001
		**IgM**	
Eotaxin	3.35	0.8 to 5.9	0.01
Fractalkine	16.9	5.7 to 28.1	0.005
IL-1α	−209.9	−410.9 to −9.0	0.04
		**IgA**	
Eotaxin	2.2	0.8 to 3.6	0.004

In HIV^+^ men, G-CSF had significant positive associations with IgG1 (*p* = 0.004) and IgG4 (*p* < 0.001) (Table 3). Adaptive cytokine IL-7 required for T cell homeostasis and development positively associated with IgG1 and IgG3 (*p* = 0.02 and *p* = 0.03, respectively) (Table 3). The levels of chemotactic, eotaxin significantly impacted total IgG (IgG1–4) in the semen (*p* = 0.005) (Table 3). Interestingly, IL-12p40 had a negative association with IgG3 (*p* = 0.01) (Table 3).

**Table 3:** Beta estimates reflecting the association between semen cytokines and Ig subclass and isotype in HIV-infected men.

**Cytokine**	**Beta Estimate ng/ml**	**Confidence Interval**	***P*-value**
		**IgG1**	
G-CSF	19.9	7.0 to 32.9	0.004
IL-7	7.1	1.1 to 13	0.02
		**IgG2**	
Eotaxin	4467.4	1423.1 to 7511.6	0.005
		**IgG3**	
IL-12p40	−515.7	−902.9 to −128.5	0.01
MIP-1α	58.00	29.2 to 86.8	<0.001
IL-7	2.6	0.3 to 5	0.03
		**IgG4**	
G-CSF	1.9	1.1 to 2.7	<0.001
RANTES	0.5	0.2 to 0.9	0.003
		**IgM**	
MCP-1	0.60	−0.02 to 1.2	0.06
		**Total IgG**	
Eotaxin	4920.6	1563.8 to 8277.4	0.005

## Discussion

In this study, we found that certain cytokine profiles in the MGT ejaculates and HIV infection status were associated with the quality and quantity of antibody responses in semen. HIV-uninfected men had distinct semen antibody isotypes, subclasses, and cytokine profiles compared to HIV-infected men. Although the titres/magnitudes of total IgG1–IgG4 were not different in HIV-infected and uninfected men's seminal fluids, the individual quantities of antibody subclasses (IgG1and IgG3) and IgM were different.

The elevated mucosal IgM, IgG1, and IgG3 may reflect the local immunity in response to HIV infection, however in the absence of functional data we cannot confirm their role in local viral control. Other studies have demonstrated the superior polyfunctionality of circulating IgG1 and IgG3 in elite controllers compared to viraemic subjects, underscoring the roles of these subclasses in viral control (49, 50). In men at risk for HIV infection, IgM, IgG1 and IgG3 demonstrated higher permeability to inner foreskin epidermis of the penis (51) whereas, HIV specific-IgA mediated neutralization was a correlate of protection in highly exposed seronegative compared to healthy, unexposed men (52). Further studies can exploit the polyfunctionality of purified IgGs and IgM in the semen of HIV infected and uninfected men to determine if these subclass or isotype profiles can be harnessed or fine-tuned to protect vulnerable mucosal surfaces.

The similar magnitudes of the IgG subclasses in semen of HIV-infected men using ARVs vs. those who were treatment-naïve, suggests that ARVs themselves do not impact the classes of antibodies that transudate into or are locally produced in the semen. Antibody isotypes and subclasses correlated significantly between the blood and semen (43, 53) and likely persists in the presence of ARVs. Even, the HIV-specific antibody activities were similar in HIV-infected men irrespective of ARV treatment. In the HIV negative men however, two men had detectable HIV-antibody specific activities to p66 in the semen, possibly indicative of HIV-exposure (54). Indeed one of these men also had mucosal antibody specific p24 responses. Previous studies have observed gag-specific immune responses in the semen in HIV-exposed seronegative, uninfected individuals (55). Despite more than half of the HIV-uninfected men having an HIV-infected partner, the cross-sectional study design presents several limitations to assessing sexual exposure to HIV. Data is lacking regarding the length of time in the serodiscordant relationship/s, viral loads of the sexual partner/s, number of concurrent sexual partners, other STI's, history of unprotected sex or condom use, circumcision and their or their partners' exposure to or use of PrEP/ARVs, respectively.

The direct associations of Env-specific antibodies gp41 and gp120 to total IgG in the semen of both HIV-infected ARV treated and ARV naïve men suggests that Env-specific antibody responses were not affected by ARV use. However, the strong and direct correlations for p24 and p66 with total IgG suggest that, in the presence of ARVs at least, transudation or even local production of certain antibodies may be affected. These discrepancies in the HIV-specific antibody profiles may suggest that ARVs may play a role in preserving specific immune responses. Although, previous studies have established that ARVs modulate the avidity of HIV-specific antibodies in patients with primary HIV-infections irrespective of the class of drugs used (56–58), others have demonstrated that ARVs did not affect antibody binding or maturation kinetics (59, 60). Whether ARV treatment directly influenced antibody titres and in turn, humoral immune functions in the MGT remain to be determined. Our data suggests that total IgG in the genital tract, may be a good proxy for titres of HIV-specific antibodies even in HIV-infected men on ARV treatment.

Our data show that inflammatory cytokines: TNFα and MIP-1β have direct associations with HIV-specific antibodies p24 and gp120 in semen of HIV-infected men. In the presence of ARVs, negative associations were found between p66 antibody specific activity and IL-6; and gp41 antibody specific activity and MIP-1α. Significant decreases in circulating as well as local genital tract pro-inflammatory cytokines were found with ARV use (61). Consistent with other studies, our data indicate that ARVs may modulate certain cytokines that in turn may affect the titres and specificities of antibodies that transudate or are locally produced in the genital tract. Even though the seminal compartment is an immunologically privileged site where local, active, viral replication can persist, even in the presence of ARVs (22, 23), HIV-specific antibody titres can be affected in the genital tract. However, the effect of the local cytokine milieu on the quality and functions of antibodies and the impact of ARVs on genital tract inflammation and immunity remain less well-defined.

Cytokines affect Ig isotype switching (62) and subclass synthesis (27). The magnitudes of IgG1 and total Ig were directly associated with the levels of IL-6, IL-8, and MCP-1 in the semen, with IgG1 appearing to mainly drive these associations in healthy men. Previous studies have shown that IL-6 induces chemokine (MCP-1) production (63), plays a key role in B-cell differentiation (64, 65) and enhances IgG production (27, 66). IL-8 may exert an indirect protective function as it recruits and activates neutrophils (67–69) that may trigger neutrophil-mediated phagocytosis of IgG opsonized HIV. Whether this pro-inflammatory signature in the seminal fluid of healthy men is protective against HIV acquisition, and is reflective of the general immunity in the MGT remains undefined. Further studies should be undertaken to determine if the IgG1 is also qualitatively/functionally different between healthy and HIV-infected men. In HIV-infected ARV naive men, IgM showed direct associations to TNFα, IL-6, and IL-10. These data suggest that in the absence of ARVs the increased IgM may be elicited to attenuate the inflammatory milieu during HIV infection (29, 30).

In conclusion, we provide evidence of higher IgG1, IgG3, and IgM in the genital tracts of HIV-infected men than in uninfected men, suggesting that HIV infection likely drives differential IgG subclass/isotype and functional responses. In the presence or absence of ARVs, HIV-specific antibodies were detected in genital fluids of HIV-infected men, suggesting that ARV use did not ablate antibody responses. Additionally, IL-6 and MIP-1α may modulate HIV-specific antibodies in ARV experienced men, whilst TNF-α and MIP-1β may modify the levels of certain HIV-specific antibodies in the genital tract in the background of inflammation in HIV-infected ARV-naïve men. This study sheds light on the profile of the HIV-specific antibodies, IgG subclasses and IgM related to the cytokine milieu present in the semen of HIV-infected and uninfected men. Considering the limited available information on the profiles of binding antibodies in the semen of HIV-infected men; this study highlights the complex interplay of the various immune parameters in the male genital mucosal environment that may be important in HIV vaccine and combination prevention studies.

## Author Contributions

TP participated in the design of the study, performed experiments, analysis and interpretation of data, manuscript writing, proof reading and final approval. PS and AO participated in analysis and interpretation of data, manuscript writing, proofreading and final approval. KN performed experiments and together with MM participated in the analysis of data. LL participated in analysis and interpretation of data. SN participated in analysis and interpretation of data. J-AP and CB participated in analysis and interpretation of data and proof reading of the manuscript. DA carried out the conception, designed and received funds to carry out the study, interpreted and analyzed the data and supervised the writing and final approval of the manuscript.

### Conflict of Interest Statement

The authors declare that the research was conducted in the absence of any commercial or financial relationships that could be construed as a potential conflict of interest.
