# Supplementary material for: Semen IgM, IgG1, and IgG3 Differentially Associate With Pro-Inflammatory Cytokines in HIV-Infected Men
Source: Front Immunol. 2019 Jan 23;9:3141. doi: 10.3389/fimmu.2018.03141 (PMC6351442; doi:10.3389/fimmu.2018.03141)
Supplement: Supplementary file 1 [file Table_1.DOCX]

**SUPPLEMENTARY TABLE 1**: Semen cytokine associations with HIV-specific antibodies in HIV^+^ARV^-^ (n=25) men.

| Cytokines | p24 | | | | p66 | | gp41 | | gp120 | |
| --- | --- | --- | --- | --- | --- | --- | --- | --- | --- | --- |
|  | **r-value** | | **p-value** | | **r-value** | **p-value** | **r-value** | **p-value** | **r-value** | **p-value** |
| IL-1**α** | 0,12 | | 0,57 | | -0,01 | 0,96 | 0,08 | 0,70 | 0,09 | 0,67 |
| IL-1**β** | 0,31 | | 0,13 | 0,16 | | 0,44 | 0,29 | 0,15 | 0,26 | 0,20 |
| IL-6 | 0,25 | | 0,23 | 0,12 | | 0,55 | 0,09 | 0,66 | 0,19 | 0,36 |
| IL-8 | 0,07 | | 0,75 | 0,06 | | 0,76 | -0,04 | 0,83 | 0,24 | 0,26 |
| IL-12p40 | 0,27 | | 0,20 | 0,14 | | 0,51 | 0,39 | 0,06 | 0,24 | 0,25 |
| IL-12p70 | -0,25 | | 0,23 | -0,28 | | 0,18 | -0,20 | 0,35 | -0,06 | 0,78 |
| TNF**α** | **0,48** | | **0,01** | 0,36 | | 0,08 | 0,36 | 0,07 | **0,44** | **0,03** |
| Eotaxin | -0,15 | | 0,49 | -0,15 | | 0,48 | -0,20 | 0,34 | 0,01 | 0,97 |
| Fractalkine | -0,09 | | 0,67 | -0,09 | | 0,67 | -0,09 | 0,65 | 0,05 | 0,81 |
| G-CSF | 0,19 | | 0,35 | 0,08 | | 0,69 | 0,20 | 0,35 | 0,18 | 0,39 |
| GM-CSF | | 0,19 | 0,37 | 0,18 | | 0,39 | 0,06 | 0,76 | 0,06 | 0,76 |
| MCP-1 | | -0,05 | 0,83 | -0,01 | | 0,96 | -0,12 | 0,58 | 0,03 | 0,90 |
| MIP-1**α** | | 0,26 | 0,21 | 0,13 | | 0,53 | 0,21 | 0,32 | 0,19 | 0,35 |
| MIP-1**β** | | **0,41** | **0,04** | 0,26 | | 0,21 | 0,30 | 0,15 | 0,35 | 0,08 |
| RANTES | | -0,10 | 0,63 | -0,15 | | 0,47 | -0,09 | 0,66 | -0,11 | 0,60 |
| IFN-g | | -0,01 | 0,97 | 0,01 | | 0,96 | -0,08 | 0,71 | -0,01 | 0,95 |
| IL-2 | | 0,20 | 0,35 | 0,15 | | 0,47 | 0,26 | 0,21 | 0,25 | 0,23 |
| IL-7 | | -0,08 | 0,71 | -0,13 | | 0,54 | -0,07 | 0,73 | -0,03 | 0,90 |
| IL-15 | | 0,01 | 0,95 | -0,02 | | 0,92 | -0,02 | 0,92 | 0,14 | 0,51 |
| IL-10 | | 0,11 | 0,61 | 0,05 | | 0,81 | 0,10 | 0,64 | 0,09 | 0,66 |
