# Supplementary material for: Semen IgM, IgG1, and IgG3 Differentially Associate With Pro-Inflammatory Cytokines in HIV-Infected Men
Source: Front Immunol. 2019 Jan 23;9:3141. doi: 10.3389/fimmu.2018.03141 (PMC6351442; doi:10.3389/fimmu.2018.03141)
Supplement: Supplementary file 2 [file Table_2.DOCX]

**SUPPLEMENTARY TABLE 2.** Semen cytokine associations with HIV-specific antibodies in HIV^+^ARV^+^ (n=11) men.

| Cytokines | p24 | | p66 | | gp41 | | gp120 | |
| --- | --- | --- | --- | --- | --- | --- | --- | --- |
|  | **r-value** | **p-value** | **r-value** | **p-value** | **r-value** | **p-value** | **r-value** | **p-value** |
| IL-1**α** | -0,44 | 0,18 | -0,33 | 0,33 | -0,36 | 0,27 | 0,11 | 0,75 |
| IL-1**β** | -0,26 | 0,43 | -0,25 | 0,47 | -0,27 | 0,42 | 0,10 | 0,78 |
| IL-6 | -0,46 | 0,15 | **-0,65** | **0,03** | -0,44 | 0,18 | -0,16 | 0,63 |
| IL-8 | -0,44 | 0,18 | -0,53 | 0,10 | -0,32 | 0,34 | -0,04 | 0,92 |
| IL-12p40 | -0,05 | 0,87 | 0,04 | 0,91 | -0,48 | 0,13 | -0,10 | 0,75 |
| IL-12p70 | -0,14 | 0,69 | -0,19 | 0,58 | -0,10 | 0,78 | 0,15 | 0,65 |
| TNF**α** | -0,42 | 0,20 | -0,50 | 0,12 | -0,43 | 0,19 | -0,02 | 0,97 |
| Eotaxin | -0,22 | 0,52 | 0,06 | 0,86 | 0,26 | 0,43 | 0,41 | 0,21 |
| Fractalkine | -0,42 | 0,20 | -0,25 | 0,47 | 0,11 | 0,75 | 0,35 | 0,29 |
| G-CSF | -0,28 | 0,40 | -0,10 | 0,78 | -0,40 | 0,23 | 0,26 | 0,43 |
| GM-CSF | -0,18 | 0,60 | -0,47 | 0,15 | -0,23 | 0,50 | -0,13 | 0,71 |
| MCP-1 | -0,29 | 0,39 | -0,18 | 0,60 | 0,08 | 0,82 | 0,24 | 0,49 |
| MIP-1**α** | -0,50 | 0,12 | -0,33 | 0,33 | **-0,73** | **0,01** | -0,09 | 0,80 |
| MIP-1**β** | -0,26 | 0,43 | -0,34 | 0,31 | -0,37 | 0,26 | -0,01 | 0,99 |
| RANTES | -0,13 | 0,71 | -0,24 | 0,49 | 0,14 | 0,69 | 0,20 | 0,56 |
| IFN-g | -0,08 | 0,82 | -0,22 | 0,52 | -0,09 | 0,80 | 0,22 | 0,52 |
| IL-2 | 0,08 | 0,82 | 0,05 | 0,88 | -0,03 | 0,95 | 0,24 | 0,49 |
| IL-7 | -0,14 | 0,69 | -0,17 | 0,61 | -0,09 | 0,80 | 0,26 | 0,43 |
| IL-15 | -0,22 | 0,52 | 0,13 | 0,71 | 0,23 | 0,50 | 0,57 | 0,07 |
| IL-10 | -0,40 | 0,23 | -0,28 | 0,40 | -0,09 | 0,80 | 0,22 | 0,52 |
