# Supplementary figures and images for: Semen IgM, IgG1, and IgG3 Differentially Associate With Pro-Inflammatory Cytokines in HIV-Infected Men
Source: Front Immunol. 2019 Jan 23;9:3141. doi: 10.3389/fimmu.2018.03141 (PMC6351442; doi:10.3389/fimmu.2018.03141)

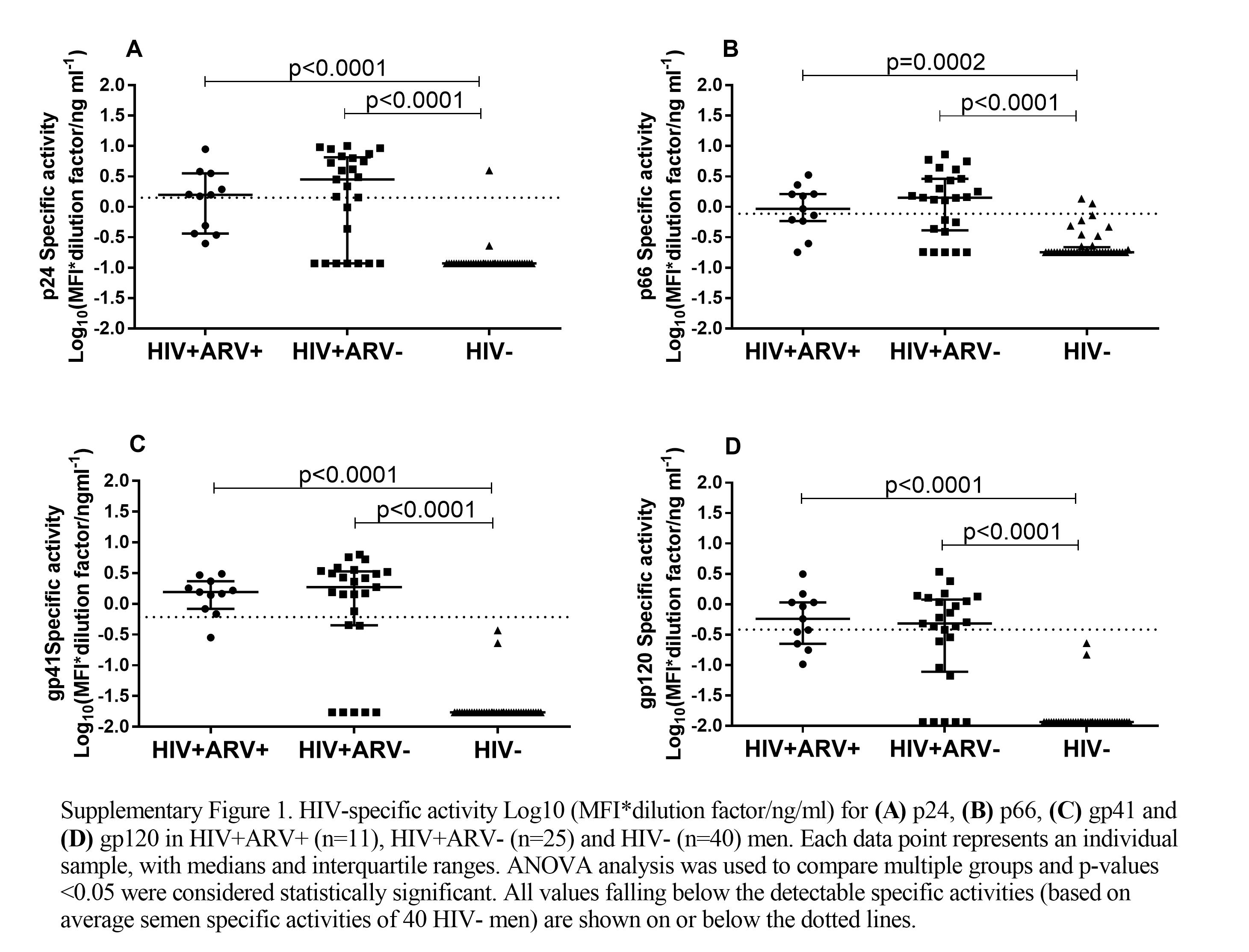

Supplement: Supplementary file 3 [file Image_1.jpg]
